# Cognitive trajectories in Parkinson’s disease patients, a review on the impact of subthalamic deep brain stimulation (STN-DBS) and emerging adaptive strategies

**DOI:** 10.1038/s41398-026-04013-6

**Published:** 2026-04-10

**Authors:** Viviane Almeida, Damian M. Herz, Jenny Blech, Matthias Hülser, Joachim Oertel, Daniel Martens, Gabriel González-Escamilla, Sergiu Groppa

**Affiliations:** 1 Department of Neurology, Saarland University, Homburg, Germany; 2 Department of Neurology, University Hospital Heidelberg, Heidelberg, Germany; 3 Department of Neuroimaging Center, University Medical Center of the Johannes Gutenberg-University Mainz, Mainz, Germany; 4 Department of Neurology, Saarland University Medical Center, Homburg, Germany; 5 Department of Neurosurgery, Saarland University Medical Center, Homburg, Germany

**Keywords:** Diseases, Neuroscience, Learning and memory

## Abstract

While deep brain stimulation (DBS) is well-established for managing motor symptoms in Parkinson’s disease (PD) and improving patient’s quality of life, its impact on cognition is still not properly addressed. Cognitive impairment in PD often affects verbal fluency, attention, and executive functions, and may interact with disease progression, dopaminergic medication, and stimulation parameters. These outcomes are shaped not only by the stimulation itself but also by factors such as baseline cognitive status, lead location, disease heterogeneity, and parameter selection. The emerging development of closed-loop DBS (CL-DBS) offers a promising tailored neuromodulation strategy that may help reduce DBS side effects while enhancing non-motor function. Given the extensive yet heterogeneous literature on cognitive outcomes after STN-DBS, this review integrates mechanistic insights from human and animal studies to clarify how stimulation parameters, anatomical targeting, and network-level dynamics influence postoperative cognitive trajectories. We summarize established cognitive effects of conventional DBS, emphasize sources of interindividual variability, and evaluate how adaptive stimulation paradigms may modulate cognitive and decision-making processes. Finally, we outline translational considerations for biomarker development and personalized neuromodulation strategies aimed at preserving cognition while maintaining motor benefit.

## Introduction

Parkinson’s disease (PD) is the second most common neurodegenerative disorder, affecting approximately 6 million individuals worldwide [1]. The Global Burden of Disease study from 2017 projected a significant increase in PD prevalence, with a 64% rise from 1990 to 2017, surpassing the growth observed in Alzheimer’s and other dementias [2].

PD arises from an interaction between genetic susceptibility and environmental or toxic impacts, beginning years before a definitive diagnosis, affecting multiple neuromodulatory systems and a broad spectrum of symptoms [3, 4]. Although clinically defined by motor symptoms as tremors, bradykinesia, and rigidity, PD presents as a heterogeneous disorder and manifests with non-motor symptoms that can significantly affect patients’ well-being, such as cognitive impairment, depression, apathy, sleep disorders, and constipation [5].

Among non-motor symptoms, cognitive impairment is a particularly relevant symptom and up to six times more common in PD than in the general population. Cognitive deficits can range from mild cognitive impairment (MCI) to severe forms of dementia, and they can strongly affect daily functioning, autonomy, and social interactions [6, 7]. Managing these symptoms, along with motor function, remains a central challenge in PD treatment.

Available therapeutic options for PD primarily focus on controlling motor symptoms and improving overall well-being. These therapies can be categorized into pharmacological and non-pharmacological interventions [8]. Dopamine replacement is the primary pharmacological treatment for managing motor symptoms. Still, it does not address the broad aspect of PD symptoms, and patients often develop motor fluctuations after several years of use [8].

Among non-pharmacological options, deep brain stimulation (DBS) is an effective and safe therapy for managing motor fluctuations, dyskinesias, or medication-resistant tremors [9]. Through the implantation of electrodes into specific brain regions, such as the subthalamic nucleus (STN) or the globus pallidus interna (GPi), connected to a neurostimulator device, DBS can modulate abnormal neuronal activity, thereby alleviating motor symptoms and enhancing the quality of life [10]. STN is frequently chosen as a target due to its strong and consistent motor benefits. Compared to other targets, STN-DBS often enables greater management of motor symptoms, reduction of dopaminergic medication and the favorable long-term clinical outcomes [11].

Despite the motor benefit, DBS’s effects on cognition remain heterogeneous in current reports, with studies differing in methodology, timing and outcome measures [12]. Nonetheless, findings from the two last decades help anchor the field. Across randomized trials, cohort studies and meta-analysis, a decline in verbal fluency consistently emerges as the most reproducible cognitive effect of STN-DBS [13–15]. Executive functions may show mild or transient changes [16]. Additionally, factors including disease heterogeneity and disease duration, but also biological individual factors such as age, sex, and baseline cognitive function may influence DBS outcomes [17, 18].

As cognitive impairment is a central and often progressive non-motor feature of PD, understanding how neuromodulation strategies may influence cognitive trajectories is important for comprehensive disease management. The field of neuromodulation is constantly evolving, and researchers have begun to shift from conventional DBS (cDBS), also known as open-loop DBS, to explore the potential of closed loop DBS (CL-DBS), referred to as adaptative DBS (aDBS), in optimizing therapeutic outcomes. Unlike cDBS, CL-DBS systems adjust stimulation parameters in real-time based on predefined electrophysiological activity, potentially minimizing side effects, reducing DBS hardware burden, and even positively modulating non-motor symptoms, including cognitive functions [19].

In the current review, we first present evidence of existing studies to synthesize mechanistic links between stimulation protocols, circuit dynamics, and cognitive domains; we then provide insights into how conventional open‑loop paradigms and CL-/tailored DBS strategies may exert cognitive effects; and finally propose measurable readouts and research designs for translation, providing practical recommendations, and a clear research agenda that bridges animal models and human physiology. Although the focus is on Parkinson’s disease, relevant findings from atypical Parkinsonian syndromes are included when they help clarify mechanistic principles or cognitive pathways. The literature review was performed by searching on PubMed and google scholar the terms: “Parkinson’s disease”, “deep brain stimulation”, “DBS”, “subthalamic nucleus”, “cognitive impairment”, “cognitive domains”, “non-motor-symptoms”, “closed-loop DBS”.

### State-of-the-art for managing PD using cDBS

Under physiological conditions, the basal ganglia, including the STN, are key contributors to motor control, supporting movement initiation, invigoration, and reinforcement, with activity patterns strongly influenced by neurotransmitters, most notably dopamine [20–22]. In PD, dopamine depletion shifts the regular pattern of STN activity into abnormal burst firing and exaggerated oscillatory activity [23, 24], which has been suggested to contribute to motor impairment [23–25].

Beyond its motor role, the STN is also involved in cognitive and limbic functions. The ventromedial STN regulates behaviors related to emotion, motivation, and reward [26], and its interactions with prefrontal cortex and other cortical areas indicate a role in decision-making, attention, and executive function [27, 28]. These cognitive processes may also be modulated by other intrinsic factors, including STN’s anatomical, molecular, and cellular status [29, 30].

High-frequency stimulation of the STN ( ~ 130 Hz) modifies membrane potentials and modulates abnormal oscillatory activity patterns at both local and network levels [31]. Thus, DBS can restore balance in basal ganglia activity, impact motor and cognitive networks, and robustly alleviate motor impairment in people with PD [30–33].

Another common target for DBS is the globus pallidus internus (GPi), a component of the basal ganglia network that also has clinical efficacy in managing the motor symptoms of PD [34–36]. GPi-DBS is considered more suited for patients with significant non-motor symptoms due to its more favorable profile concerning cognitive and mood-related side effects than STN [33, 37–39]. Yet, some studies report no substantial cognitive differences between targets; therefore, STN-DBS is commonly chosen for its superior motor symptom relief, a substantial reduction in medication requirements, and potential for longer battery life [32, 36, 40, 41].

### Targets and stimulation parameters

DBS efficacy depends not only on stimulation itself but also on the precision of anatomical targeting. Small variations in lead placement can influence motor benefit and generate unintentional effects on cognition and mood [42, 43]. Anteromedial STN implantation had been associated with hypomania in some patients [30]; implantation near the associative regions can impact cognitive function. Areas closely connected to the prefrontal cortex can affect executive function; moreover, the proximity of the electrodes to the limbic regions of the STN has been associated with mood disturbances and verbal fluency [44–47].

Recent advances in neuroimaging and intraoperative mapping have improved the precision of electrode placement [48, 49], mitigating small deviations that could impact quality of life and allowing for a personalized DBS therapy that optimizes motor control while preserving other functions [42].

In addition to anatomical targeting, the efficacy and side effects of DBS can also be influenced by the stimulation parameters applied. While high-frequency stimulation is typically used for motor symptom control in PD, different frequency ranges may engage cognitive or affective circuits. Low-frequency DBS of the fornix ( ~ 20 Hz) in Alzheimer’s disease has shown improvements in memory, likely by modulating the Papez circuit and the default mode network [50]. In contrast, frequency stimulation ( > 100 Hz) of the anterior thalamic nucleus (ANT), has been associated with cognitive side effects, including memory decline in epilepsy patients [51, 52].

### Cognitive effects after STN-DBS

Concerning cognition, reports on the impact of STN-DBS are mixed. Cognitive decline in PD affects multiple domains, including verbal fluency, memory, attention, and executive functions, manifesting both before and after clinical diagnosis. Despite these impairments, many PD patients report improved quality of life and reduced depressive symptoms after DBS [37].

### Short and long-term effects on cognitive domains

Multiple studies have reported declines in specific cognitive domains after STN-DBS, particularly in verbal fluency and memory [53–55]. Tröster [38] synthesized 15 standardized neuropsychological studies (n > 600) and found consistent, moderate post-DBS declines in verbal fluency and executive function. Zangaglia et al. [16] observed transient executive dysfunction within the first year post-surgery in a three-year follow-up study. In a six-year observational study, 41% of STN-DBS patients developed MCI or dementia, with pronounced decline in global cognition, executive function, and verbal fluency [56]. Complementary evidence from a matched case-control study confirmed domain-specific cognitive changes following STN-DBS, including declines in verbal fluency, memory, and visuospatial abilities. Importantly, the study noted that these effects remained significant even after adjusting for demographic and disease-related factors, reinforcing the notion that a subset of patients may experience domain-specific cognitive deterioration despite stable or improved motor outcomes [57].

Arten and Hamdan [58] studied the impact of demographic factors on the cognitive performance of PD patients undergoing DBS. They found significant differences in executive functions and memory, with poorer cognitive performance in the DBS group compared to non-surgical controls.

A meta-analysis by Bucur & Papagno [37] reported long-term decrease in memory, phonemic fluency, and specific subdomains of executive functions. Wang et al. [59] further confirmed persistent impairments in semantic and phonemic fluency at 6 and 12 months post-surgery, despite stabilization or improvement in other domains.

A large randomized controlled trial comparing STN-DBS with best medical therapy further confirmed this pattern: despite significant motor improvement, patients showed selective declines in semantic and phonemic fluency, as well as reduced performance in interference-control tasks, while global cognition, memory, and attention remained stable. Importantly, anxiety levels improved, and depressive symptoms did not worsen, reinforcing the interpretation that cognitive effects are domain-specific rather than globally deteriorative [13]. Changes in decision-making have also been reported after STN-DBS. Although not routinely evaluated in standard cognitive batteries, alterations in response caution, impulsivity, and decision thresholds have been observed, particularly in tasks involving conflict or time pressure. These changes may reflect the STN’s role in inhibitory control and integration of cognitive and motor demands [28, 60, 61].

### Variability and stability in cognitive outcomes

In many patients, cognitive effects stabilizes over time, and global cognitive function may not deteriorate significantly, suggesting that long-term outcomes may be mild in some cases [62–64]. In a randomized clinical trial Hacker et al. [65] compared PD patients receiving STN-DBS plus optimal drug therapy (ODT) to those receiving ODT alone. Over a five- and eleven-year follow-up, the study found no significant long-term cognitive decline in the DBS group compared to controls, with initial declines in verbal fluency and processing speed diminished over time.

**Fig. 1:**
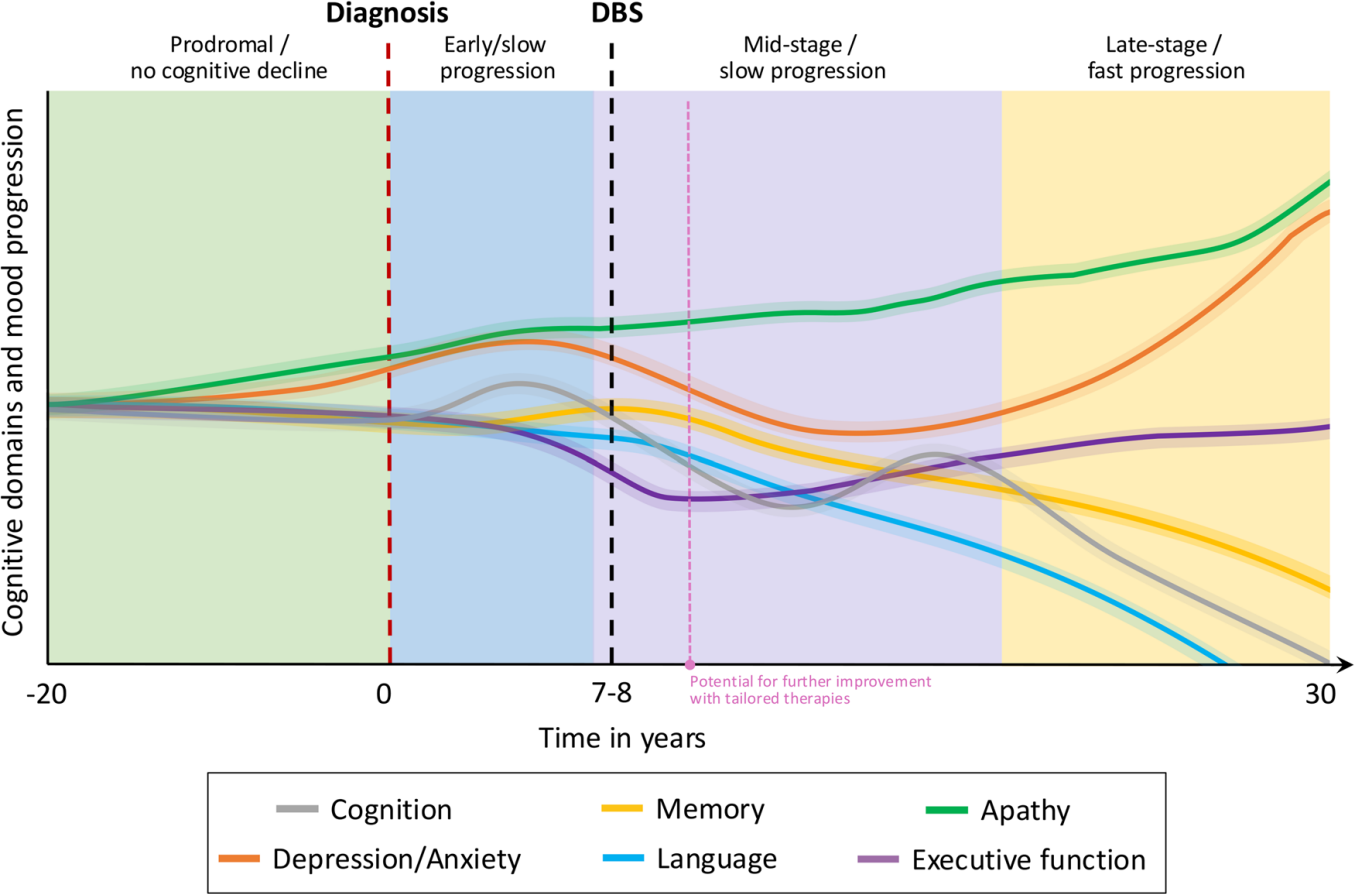
Schematic representation of domain-specific cognitive and mood trajectories across Parkinson’s disease progression and after STN-DBS. The figure summarizes reported patterns in cognitive domains and mood symptoms over time. While early stages show relative stability, variability increases in the mid-phase. After DBS, mild domain-specific effects may occur, with some recovery (e.g., executive function) and others showing progressive decline (e.g., memory, language). Mood symptoms may initially improve postoperatively but tend to worsen over time. The magenta marker indicates the potential for further improvement with tailored interventions such as CL-DBS. Shaded areas reflect inter-individual variability.

These findings suggest that long-term cognitive trajectories are thought to primarily reflect underlying PD progression rather than direct DBS effects (Fig. 1). Age, baseline cognition, disease duration, and non-dopaminergic therapies all influence cognitive outcomes [38, 66]; older patients are more likely to experience cognitive deterioration post-DBS, and pre-existing mild cognitive impairment (MCI) increases the risk of further cognitive decline [6, 67, 68], underscoring the importance of patient selection.

Consistent with this, large-cohort evidence from Rothlind et al. [66] showed that multidomain cognitive decline after STN-DBS is relatively uncommon and is largely driven by age and lower baseline cognitive performance, reinforcing the view that postoperative cognitive changes reflect individual vulnerability rather than stimulation-related effects.

Recent research has also suggested that patients with moderate cognitive impairment may still benefit from motor improvement without a higher risk of adverse events, similar to individuals with normal cognition [6]. Nonetheless, long-term observations are needed to investigate the risk of faster cognitive deterioration and/or disease progression. (Table 1)

**Table 1:** Overview of Key Studies on Cognition and STN-DBS.

Reference	Scope	Key topics covered	Main conclusions on cognition	Gaps & relevance
Racki et al. [64]	Systematic review of cognitive outcomes after DBS in PD.	Global cognition; fluency; EF; memory; target comparisons.	Shows a pattern of preserved global cognition with selective vulnerability of fluency, suggesting domain-specific rather than global cognitive impact.	Highlights inconsistent methodology and lack of parameter–cognition analyses, underscoring the need for mechanistic frameworks linking settings to domain-specific effects.
Guidetti et al. [139]	Review on adaptive DBS (aDBS) across motor, cognitive, and psychiatric disorders.	Biomarkers; sensing; adaptive control; frequency-specific modulation.	Suggests that aDBS has theoretical potential to shape cognitive control circuits but provides no clinical evidence.	Emphasizes the absence of validated biomarkers and cognitive endpoints, supporting the need for translational models connecting stimulation dynamics and cognitive outcomes.
Witt et al. [13]	RCT evaluating neuropsychological effects of STN-DBS.	Executive function, verbal fluency, psychiatric symptoms.	Demonstrates selective fluency decline with otherwise stable cognition, reinforcing the idea of focal, not global, cognitive susceptibility.	Limited follow-up and no anatomical/parameter analyses; illustrates early evidence of domain-specific cognitive costs of STN-DBS.
Cabrera-Montes et al. [11]	9-year prospective matched-controlled STN-DBS study.	Motor outcomes; global cognition; fluency; EF; memory.	Long-term stability of global cognition with persistent fluency decline reinforces a chronic, domain-specific pattern.	Few long-term controlled cohorts; mechanisms behind fluency drop remain unclear; emphasize the need for parameter- and circuitry-level explanations.
Cole et al. [134]	Experimental study comparing theta vs high-frequency STN-DBS during cognitive control.	STN theta; decision thresholds; DDM; conflict processing.	Demonstrates frequency-dependent cognitive modulation: theta stimulation enhances deliberation, contrasting with impulsivity under high-frequency DBS.	Short-term design and limited generalizability; supports emerging models where specific DBS parameters selectively shape cognitive control.
Tröster, [38]	Narrative review focused on predictors of cognitive change after DBS (2018–2024).	Predictive factors; patient selection; neuropsychological profiles; surgical/stimulation variables.	Synthesizes evidence that cognitive decline after DBS is generally mild and domain-specific, with age and pre-op cognitive status emerging as the most consistent predictors.	Points to the lack of strong predictive models, the heterogeneity of available studies, and the insufficient integration of imaging and stimulation data. Reinforces the need for mechanistic, parameter-level approaches.
Longo et al. [57]	Case–control study using MCID to assess cognitive change after STN-DBS.	Cognitive domains (memory, attention, visuospatial, fluency); MCID; matched controls.	Identifies subtle, domain-specific changes with preserved global performance; supports selective cognitive susceptibility.	Underscores the importance of consistent pre-op cognitive profiling, rigorous controlled designs, and integration of stimulation settings. Reinforces the move toward more accurate prediction frameworks for DBS-related cognition.
Wolters et al. [118]	Narrative review on cognitive effects of classical and novel STN-DBS paradigms.	STN circuitry; classical DBS cognitive outcomes; theta/alpha/gamma oscillations; novel paradigms.	Argues cognitive effects depend on associative/limbic pathways and oscillatory tuning; supports frequency-specific frameworks.	Notes the fragmented evidence base for novel stimulation paradigms and the limited mechanistic mapping between parameters, oscillations, and cognitive effects. Underscores the need for circuit-based, parameter-level frameworks.
Schor et al. [151]	Mechanistic study of STN-DBS using artifact-free calcium imaging and optogenetics in parkinsonian mice.	STN/SNr firing; hyperdirect pathway; movement-related dynamics; optical DBS; parameter effects.	Demonstrates that DBS disrupts pathological STN patterns rather than inhibiting firing; supports a timing-based mechanism over rate-based or hyperdirect-driven models.	Lacks cognitive behavioral paradigms and higher-order assessments; findings restricted to motor circuits and rodent STN physiology. Highlights need to extend circuit-level insights to cognitive domains and to human-relevant stimulation parameters.
Rothlind et al. [66]	Predictors of multidomain cognitive decline after STN-DBS.	Neuropsychiatric assessment; risk modelling; decline trajectories.	Shows decline is infrequent and largely explained by age + baseline cognitive status; supports patient-level vulnerability model.	Lack of mechanistic insights; supports need for individualized prediction models.
Planche et al. [105]	Anatomical predictors of post-DBS cognitive decline.	Lead position; structural markers; postoperative neuropsychology.	Ventral/limbic STN spread and atrophic patterns predict cognitive worsening, particularly in fluency and executive domains.	Older methodology; no connectivity or modern imaging; foundational but needs high-resolution connectivity data.
Bucur & Papagno, [37]	Meta-analysis of long-term neuropsychiatric outcomes after STN-DBS.	Long-term cognition; verbal fluency decline; domain-specific effects.	Verbal fluency decline is consistent; other domains remain relatively preserved; heterogeneity across studies persists.	Meta-analysis limited by old datasets; highlights need for modern, mechanistic, and parameter-sensitive cognitive research.
Sisodia et al. [74]	Systematic review on DBS–cognition interactions.	Bidirectional mechanisms; STN functional domains; parameters; tasks; tractography.	Cognition is modulated by stimulation site, frequency, and state; cognitive outcomes reflect network dynamics rather than simple “side effects.”	No quantitative synthesis; emphasizes need for mechanistic studies.
Del Bene et al. [127]	Cognitive effects of unilateral STN-DBS.	Hemisphere effects; directional vs ring; verbal fluency; inhibition.	Shows left STN worsens fluency while right STN may improve inhibition; supports laterality-dependent cognitive effects.	Short follow-up; unilateral only. Provides evidence that cognition depends on laterality and network targeting, not just DBS itself.
Salehi et al. [125]	Theta STN-DBS and working memory.	Theta STN stimulation; DLPFC–STN connectivity.	Provides direct evidence that theta DBS enhances working memory; strong support for oscillation-specific modulation.	Small sample; experimental setting. Strongly supports the mechanistic angle that cognitive effects depend on frequency-dependent modulation of STN–PFC loops.

### Mechanisms and management of outcome variability

Well-managed motor symptoms through DBS could also have a positive impact on cognition when patients’ perception of treatment outcomes is positive. Given the burden of PD symptoms, improvements can enhance quality of life and due to subjective expectation, reduce cognitive load [69, 70].

Beyond stimulation-related effects, the surgical trajectory itself may also influence cognitive outcomes. Although most cognitive effects are attributed to chronic stimulation, there is evidence that some patients experience acute and sustained declines in tasks related to frontal executive control following electrode implantation, independent of stimulation status [71–73].

Longitudinal studies also provided further insights into the cognitive outcomes of STN-DBS. A 12-month follow-up study conducted by Liang et al. [12] reported non-permanent cognitive outcomes. Although motor symptoms, anxiety, and depression improved significantly by the 12-month follow-up, the patient’s performance on cognitive tests showed no significant change from baseline. This was further supported by a two-year follow up study from Hong et al. [53] of young-onset PD patients with STN-DBS, that found an stable overall cognitive performance, and both depression and anxiety symptoms significantly improved.

Sisodia et al. [74] conducted a systematic review and meta-analysis exploring the connection between cognition and DBS in PD patients. Although they found a moderate decline in verbal fluency, it was not permanent; other cognitive domains, such as memory and executive function, showed no significant long-term changes.

The success of current chronic high-frequency STN-DBS paradigms for both motor and non-motor symptoms may be improved by the meticulous patient selection, considering age, disease progression, pre-existing disease conditions, surgical precision, and postoperative parameter adjustments. DBS programming, for example, can be challenging since it remains a trial-and-error method based on clinical observations [75]. Therefore, approaches that offer a tailored and responsive treatment option addressing the limitations of standard DBS are needed, and the emergence of adaptive studies offers the possibility of overcoming these limitations.

### Further factors affecting cognitive performance beyond DBS

Although some cognitive changes after DBS arise from surgical or stimulation-related mechanisms, several factors unrelated to the stimulation itself substantially shape cognitive trajectories in PD [64]. These sources of variability provide important context for interpreting postoperative outcomes and for understanding why similar DBS protocols give different cognitive profiles across patients [66].

### Disease heterogeneity

PD encompasses multiple phenotypic and progression subtypes that influence baseline cognitive vulnerability. Non-tremor-dominant presentations, faster motor progression, autonomic dysfunction, and REM sleep behavior disorder have all been associated with earlier and more pronounced cognitive impairment [76–78]. The dual-syndrome hypothesis distinguishes an executive/fronto-striatal subtype from a posterior-cortical memory/visuospatial subtype, each with distinct neuroanatomical and neurochemical signatures [79]. Based on this concept, cluster-based subtyping studies also identify “diffuse/malignant” phenotypes characterized by early frontal–executive deficits and rapid global decline, suggesting that some patients enter surgery with intrinsically higher predisposition to postoperative deterioration. These frameworks highlight how patient subtyping can help identify individuals who may be more vulnerable to postoperative cognitive changes [79, 80].

### Sleep

Sleep dysfunction is closely linked to cognitive impairment in PD, through impaired attention, executive function, and memory [81, 82]. REM sleep behavior disorder, excessive daytime sleepiness, and sleep apnea consistently predict faster cognitive decline and may compound postoperative cognitive fluctuations [83–85].

### Neurodegenerative pathology and biomarkers

At a pathological level, cognitive vulnerability in PD is strongly influenced by overlapping proteinopathies. Lewy bodies pathology, driven by misfolded α-synuclein, and frequently accompanied by amyloid-β and tau pathologies, accelerates cortical neurodegeneration and is strongly associated with faster and more severe cognitive decline in PD [86–88]. Additionally, emerging plasma and cerebrospinal fluid biomarkers have been associated with cognitive susceptibility, including protein and metabolic profiles recently described, suggesting that multiple molecular pathways may contribute to the risk of cognitive decline in PD [89–91].

### Genetic influences

Recent studies showed that variants in GBA and APOE were associated with an earlier and more pronounced cognitive decline, whereas LRRK2 mutations may have a milder or even similar cognitive profile to those without mutations [77, 92–95]. Early data suggest that genetic background may modulate both baseline cognitive vulnerability and responsiveness to DBS, although evidence remains limited [96]. Other loci (e.g., SNCA, PINK1) have also been implicated, but findings are inconsistent, and their impact on cognition and DBS outcomes remains unclear [96–98].

### Aging, brain reserve, and other comorbidities

Advanced biological aging and reduced brain reserve, reflected, for instance, by increased grey- and white-matter predicted-age differences (GM-PAD and WM-PAD), have been associated with poorer cognitive performance and a higher risk of deterioration in PD [99, 100]. Systemic factors, including elevated BMI, vascular comorbidities, and metabolic markers such as homocysteine, may add to cognitive susceptibility [101–103]. Studies from longitudinal biomarkers, such as NfL, and structural measures (reduced cortical thickness and altered microstructural integrity) across several brain regions, suggest that subclinical brain vulnerability is already present before surgery and may predispose patients to postoperative cognitive deterioration [72, 104]. In line with this, structural MRI work has shown that preoperative frontal–limbic and ventral striatal atrophy predicts postoperative executive decline after STN-DBS, further supporting the role of anatomical vulnerability in shaping cognitive trajectories [105].

### Psychiatric symptoms and cognitive vulnerability

Depression, apathy, and anxiety are highly prevalent in PD and often coexist with cognitive impairment. Although these symptoms are not direct indicators of cognitive decline, they may influence test performance, increase perceived cognitive load, or reflect dysfunction of frontal-limbic circuits.

Moreover, the interaction between dopaminergic medication, DBS, and neuropsychiatric symptoms further complicates the interpretation of cognitive outcomes, highlighting the need to evaluate mood and motivation alongside cognition in the post-DBS period [106–108].

## Closed-loop DBS: advances and further potential

### Mechanistic foundations and emerging applications

Closed-loop DBS (CL-DBS) has shown safety and efficacy as an alternative to conventional open-loop DBS (cDBS), particularly in real-life settings and during periods of reduced movement, providing notable reductions in motor symptoms, while delivering substantially less electrical energy to the brain [19, 109–114]. The CL-DBS approach adjusts stimulation in real time using electrophysiological biomarkers, most commonly beta-band activity (13–30 Hz) [115]. However, recent work has expanded this landscape: theta and alpha-band activity have been linked to cognitive and affective states [116], and stimulation-entrained gamma oscillations ( ~ 65–70 Hz) have been demonstrated as stable, physiologically meaningful biomarkers for adaptive control [117]. Recent review similarly emphasized that these alternative dynamics may extend the scope of STN-DBS toward cognitive and affective modulation [118].

These observations broaden the mechanistic basis of CL-DBS beyond pathological beta suppression. Experimental paradigms have illustrated how stimulation can potentially modulate pathways, including thresholded beta suppression for motor control [119, 120], theta-triggered bursts as an experimental strategy to modulate cognitive control [28, 121] and state-dependent stimulation during ongoing motor or cognitive task engagement [28, 122].

Further supporting this shift from rate- to dynamics-based interpretations of DBS mechanisms, Schor et al. [119] showed in a mouse model that therapeutic STN-DBS disrupts movement-related STN activity rather than altering mean firing rates. Movement-locked STN patterns were abolished during stimulation, a change necessary and sufficient for motor improvement. Though not focused on cognition, these results reinforce a core principle relevant to adaptive neuromodulation: effective DBS disrupts pathological activity patterns in a state-dependent manner, supporting the rationale for timing-sensitive, biomarker-guided closed-loop strategies.

Although large-scale clinical trials such as ADAPT-PD have focused primarily on motor outcomes (showing motor benefits and reduced stimulation energy) [123], mechanistic studies indicate that biomarker-driven stimulation can influence cognitive processes. Beta-based adaptive stimulation has been shown to influence response caution during decision-making [124], while theta-frequency DBS has improved aspects of cognitive control and working memory without impairing motor performance [125].

Beyond oscillatory activity, a broad spectrum of physiological and biochemical signals is being explored as candidate markers for adaptive control. These include local field potentials (LFPs) and biochemical indicators of neurotransmission activity [126], each offering a window into dynamics changes in network state. At present, no evidence supports the idea that either cDBS or CL-DBS slows PD progression and cognitive decline still largely reflects the underlying disease course [127, 128]. Nevertheless, changes in oscillatory activity associated with motor impairment may also reflect behavioral and cognitive functions, suggesting that CL-DBS could be used to manage non-motor symptoms [129, 130].

### Cognitive and behavioral modulation

Research on CL-DBS and non-motor symptoms remains limited, but early findings suggest broad potential. A qualitative study by Merner et al. [131] explored the psychosocial impact of CL-DBS on individuals who participated in clinical trials with treatment-resistant conditions. Participants reported positive changes in personality, mood, and behavior, along with increased quality of life, with minimal and temporary adverse effects.

CL-DBS targeting motor symptoms via beta-power modulation also alters decision-slowing strategies [124]. Reductions in beta activity (13-30 Hz) have been associated with lowered decision thresholds and faster, but potentially less deliberate actions [28], whereas theta activity (2–8 Hz) appears associated with improved cognitive control and more cautious decision-making [132, 133]. Supporting this, theta-frequency (6 Hz) DBS improved working memory in PD without impairing motor impairment, likely via STN–middle frontal gyrus connectivity, a region involved in cognitive control processes [125].

Further evidence shows frequency-specific effects. Cole et al. [134] demonstrated that low-frequency (4 Hz) STN stimulation increased decision thresholds, in direct contrast to conventional ~130 Hz stimulation, providing causal evidence that low-frequency STN stimulation supports more deliberate responding and improved executive control.

### Non-motor domains beyond cognition

CL-DBS has also been investigated in sleep: multi-night intracranial recordings revealed significant cortical-subcortical interactions during non-REM sleep, with DBS enhancing cortical delta and reducing beta activity. Increases in subcortical beta preceded awakenings, suggesting a mechanism for sleep interruptions in PD. The research also showed CL-DBS’s potential to improve sleep, with high accuracy in classifying sleep stages via intracranial signals [135]. Also, a recent study provided proof-of-principle that adapting DBS frequencies to 4 Hz stimulation during NREM sleep can improve memory consolidation in PD compared to cDBS [136].

A study exploring DBS in the subcallosal cingulate (SCC-DBS) for depression illustrates how adaptive neuromodulation can modulate affective circuits, findings that may be relevant for DBS in PD patients, especially regarding non-motor domains, and that guide future research [137].

Together, these studies, though often small or experimental, underscore the expanding potential of CL-DBS. The integration of diverse biomarkers may enable progressively refined neuromodulation strategies that target motor and non-motor states with greater precision.

## Comparison between open-loop (cDBS) and closed-loop DBS (CL-DBS)

### Principles and operational differences

cDBS delivers continuous, fixed stimulation independent of behavioral or neural state, producing reliable motor improvement but often leading to over- or under-stimulation across daily fluctuations. CL-DBS introduces real-time adjustments based on physiological signals, refining when stimulation is delivered without altering the core mechanism of DBS [138].

### Mechanistic distinctions

Both approaches influence basal ganglia–thalamocortical circuits, but with different temporal precision. cDBS provides continuous nonspecific modulation, whereas CL-DBS aligns stimulation with ongoing neural dynamics. This mechanistic principle of CL-DBS therefore lies in timing and state-alignment, not in targeting different circuits than cDBS [139, 140].

### Clinical outcomes and evidence

cDBS remains highly effective for motor symptoms, supported by extensive long-term data. CL-DBS provides comparable motor improvement with lower stimulation energy and improved adaptation across behavioral states. Evidence for cognitive modulation comes from early mechanistic studies, but large trials remain motor-focused, and cognitive advantages have not yet been established in clinical endpoints [122].

### Translational implications

The transition from cDBS to CL-DBS represents a broader shift toward physiology-guided neuromodulation. Key priorities include long-term validation, multi-site biomarker standardization, and integration of adaptive algorithms into clinically scalable frameworks [139].

## Current and prospective challenges for leveraging the potential of closed-loop DBS to improve cognition

### Interindividual variability and longitudinal instability

Adaptive systems must account for individual differences in age, disease stage, subtype, comorbidities, and cognitive vulnerability. As PD progresses, cognitive decline or changes in decision-making capacity may alter responsiveness to adaptive algorithms, requiring systems that can evolve with the patient [141–143].

### Gaps between mechanistic discovery and clinical translation

Mechanistic animal studies have elucidated biomarker-guided stimulation protocols, but comprehensive frameworks for translating these findings into robust clinical trials are only now emerging [144–146]. Recent work using rodent models to test on-off and proportional adaptive DBS algorithms [141], flexible graphene-based electrodes for high-resolution stimulation and recording [147], and longitudinal electrophysiological datasets for biomarker development [143] contributes to the progress of experimental research models toward clinical application.

### Biomarker limitations

Despite extensive research and effort to identify biomarkers for PD, determining a reliable disease-progression marker remains complex, given the variability and heterogeneity of PD’s motor and non-motor trajectories. Limited sensitivity in the prodromal phase makes it difficult to pinpoint reliable, univocal biomarkers for diagnosing and tracking non-motor symptoms [148]. Preliminary studies on theta and alpha oscillations may reflect non-motor states [116], but their variability across individuals complicates implementation. Standardizing biomarker selection, validation, and interpretation remains an ongoing effort [148, 149].

Despite extensive research and effort, identifying reliable biomarkers for PD remains difficult given the heterogeneity of motor and non-motor trajectories. Limited sensitivity in the prodromal phase makes it difficult to pinpoint reliable, univocal biomarkers for diagnosing and tracking non-motor symptoms [148]. Preliminary studies on oscillatory markers may reflect non-motor states [116], but show considerable interindividual variability. Standardizing biomarker selection, validation, and interpretation remains an ongoing effort [148, 149].

### Circuit specificity and anatomical constraints

Even with adaptive algorithms, accurate anatomical targeting remains essential. Adaptive systems refine when stimulation is delivered but do not inherently distinguish between stimulation in motor or non-motor STN regions, which may increase cognitive or mood-related side effects. As such, CL-DBS does not compensate for suboptimal anatomical targeting [110, 150].

### Hardware, computational demands, and long-term evaluation

Adaptive systems require continuous sensing, real-time processing, and stable power supplies to operate effectively. Integrating multimodal data increases complexity, and patient-specific configuration remains time-consuming. Ethical considerations regarding patient autonomy in parameter adjustment add further complexity [139]. Additionally, long-term studies are necessary to evaluate the algorithm’s safety, stability, and sustained benefits, particularly for applications where effects are subtle and may shift over time. Robust follow-up protocols will be required to detect gradual changes in stimulation responsiveness [128].

## Conclusion

The complexity of PD extends beyond the predominant motor symptoms to include non-motor symptoms, such as cognitive impairment, which strongly affects independence and overall well-being. While STN-DBS reliably improves motor outcomes, its cognitive effects remain variable, influenced by disease heterogeneity, baseline cognitive status, age, and surgical targeting. Therefore, it is important to consider careful patient selection and ongoing adjustments in DBS therapy to optimize its impact on non-motor symptoms.

CL-DBS offers a promising advance by allowing real-time, biomarker-guided stimulation adjustments, which have the potential to minimize side effects and optimize therapeutic outcomes. However, the implementation also brings new challenges, including biomarker standardization, technological constraints, and variability in patient response.

Future research lies in optimizing DBS protocols, involving further investigation into reliable biomarkers, long-term studies to assess sustained benefits, and the development of adaptive algorithms. Technological refinement may facilitate the development of effective strategies for early detection, treatment that focuses on both motor and non-motor symptoms, and the delay or interruption of disease progression.
